# Changes in the tumor oxygenation but not in the tumor volume and tumor vascularization reflect early response of breast cancer to neoadjuvant chemotherapy

**DOI:** 10.1186/s13058-023-01607-6

**Published:** 2023-01-30

**Authors:** Mikhail V. Pavlov, Anna P. Bavrina, Vladimir I. Plekhanov, German Yu. Golubyatnikov, Anna G. Orlova, Pavel V. Subochev, Diana A. Davydova, Ilya V. Turchin, Anna V. Maslennikova

**Affiliations:** 1 Nizhny Novgorod Regional Clinical Oncology Dispensary, Delovaya St., 11/1, Nizhny Novgorod, Russia 603126; 2 Privolzhsky Research Medical University, Minina Square, 10/1, Nizhny Novgorod, Russia 603950; 3 Institute of Applied Physics RAS, Ul’yanov Street, 46, Nizhny Novgorod, Russia 603950; 4 National Research Lobachevsky State University of Nizhny Novgorod, Gagarin Ave., 23, Nizhny Novgorod, Russia 603022

**Keywords:** Breast cancer, Neoadjuvant chemotherapy, Diffuse optical Imaging, Ultrasound, Doppler ultrasound, Predictive criteria, Pathological tumor response, Logistic regression models

## Abstract

**Background:**

Breast cancer neoadjuvant chemotherapy (NACT) allows for assessing tumor sensitivity to systemic treatment, planning adjuvant treatment and follow-up. However, a sufficiently large number of patients fail to achieve the desired level of pathological tumor response while optimal early response assessment methods have not been established now. In our study, we simultaneously assessed the early chemotherapy-induced changes in the tumor volume by ultrasound (US), the tumor oxygenation by diffuse optical spectroscopy imaging (DOSI), and the state of the tumor vascular bed by Doppler US to elaborate the predictive criteria of breast tumor response to treatment.

**Methods:**

A total of 133 patients with a confirmed diagnosis of invasive breast cancer stage II to III admitted to NACT following definitive breast surgery were enrolled, of those 103 were included in the final analysis. Tumor oxygenation by DOSI, tumor volume by US, and tumor vascularization by Doppler US were determined before the first and second cycle of NACT. After NACT completion, patients underwent surgery followed by pathological examination and assessment of the pathological tumor response. On the basis of these, data regression predictive models were created.

**Results:**

We observed changes in all three parameters 3 weeks after the start of the treatment. However, a high predictive potential for early assessment of tumor sensitivity to NACT demonstrated only the level of oxygenation, ΔStO_2_, (*ρ* = 0.802, *p* ≤ 0.01). The regression model predicts the tumor response with a high probability of a correct conclusion (89.3%). The “Tumor volume” model and the “Vascularization index” model did not accurately predict the absence of a pathological tumor response to treatment (60.9% and 58.7%, respectively), while predicting a positive response to treatment was relatively better (78.9% and 75.4%, respectively).

**Conclusions:**

Diffuse optical spectroscopy imaging appeared to be a robust tool for early predicting breast cancer response to chemotherapy. It may help identify patients who need additional molecular genetic study of the tumor in order to find the source of resistance to treatment, as well as to correct the treatment regimen.

**Supplementary Information:**

The online version contains supplementary material available at 10.1186/s13058-023-01607-6.

## Background

Neoadjuvant chemotherapy (NACT) as the standard of care for patients with newly diagnosed breast cancer (BC) is now widely used to shrink the tumor before surgical treatment and reduce the volume of axillary lymph node dissections [1, 2]*.* This approach allows for assessing tumor sensitivity to systemic treatment, planning adjuvant treatment and follow-up [3]. Significant advances in the management of breast cancer are associated primarily with the personalization of the therapy depending on the molecular characteristics of the neoplasm [4, 5]. A pathologic complete response (pCR) to chemotherapy which means the absence of viable tumor cells in the breast tissue after surgery is a significant criterion that correlates with a favorable prognosis and good survival rates [1]. However, in case of the absence of a known molecular target, a sufficiently large number of patients fail to achieve the desired level of pathological tumor response (PTR) [1, 6]*.* Those patients may need additional genetic tests to find the cause of resistance to chemotherapy and their therapy should be altered to avoid unjustified toxicity, surgery delay and high cost of ineffective treatment [7, 8]*.* Unfortunately, optimal early response assessment methods have not been established now.

It would be attractive to use the dynamic of tumor volume as a simple and easily detectable biomarker. Standard cost-effective imaging modality ultrasound (US) investigation seems to be very proper for solving this problem, but earlier studies showed low efficacy of the method [9, 10]*.* Studies conducted in recent years demonstrated better efficacy of the US for predicting pCR but not the lack of response to treatment. The accuracy of predicting PTR to treatment depended on the cancer subtype [11–15]*.* Contrast-enhanced MRI [16] and 18F-FDG PET/CT [7, 17, 18] have demonstrated a good predicting ability by evaluation of the metabolic activity of tumor tissue. Both methods suffer from high costs and require the administration of contrast agents or radiopharmaceuticals, which limits their application for treatment monitoring.

Optical methods based on the difference in the scattering and absorption properties of normal and tumor tissues have been actively developed for diagnosis [19, 20] and monitoring of breast pathology [21–24]*.* Diffuse optical spectroscopy imaging (DOSI) utilizes light of far-red and near-infrared spectral range (~ 650 to 1000 nm), which has a low attenuation rate in tissue. The main chromophores in this range are oxyhemoglobin and deoxyhemoglobin, the concentration of which indirectly reflects oxygen delivery and consumption by tissues [25–27]*.* Evaluating their concentration, one can assess the level of tumor oxygenation (hypoxia) which appears to be an important characteristic of tumor biology [28]*.* Multiple studies have demonstrated DOSI data to be a prospective marker that reflects pathological tumor response at various times from the NACT starts [21, 24, 29–33]*.* The combined use of DOSI and assessment of the dynamics of tumor volume by the US, as well as taking into account tumor immunophenotype, made it possible to increase the predictive capabilities of the method [34]*.*

Tumor oxygenation is inextricably linked with the state of its vascular bed [35, 36]*.* Antitumor treatment can affect both the cells and the stroma of the tumor, including its microvessels [37]*.* So, according to the reaction of the tumor vascular bed, it is possible to indirectly assess the sensitivity of the neoplasm to drug exposure. Doppler ultrasound investigation is a noninvasive, widespread, and relatively inexpensive vascular imaging method, which can be safely used for repeated measurements [38]*.* Change in the number of registered vessels turned out to be the independent factor reflecting the tumor response to treatment [39–41]*.*

In our study, for the first time, we simultaneously assessed the early chemotherapy-induced changes in the tumor volume (by US), tumor oxygenation (by DOSI), and the state of the vascular bed (by Doppler US) to elaborate the predictive criteria of breast tumor response to treatment.

## Methods

### Patients’ data

The study was performed at Nizhny Novgorod Regional Clinical Oncology Hospital (Russia) from June 2016 to August 2021. A total of 133 patients with a confirmed diagnosis of invasive breast cancer stage II to III admitted to NACT following definitive breast surgery were enrolled, and 108 of them completed the therapy. Patients with tumors larger than 8 cm, fungating breast cancers, and age under 18 were excluded. Patient informed consent and study methods were reviewed by the local Research Ethics Border.

### Study design

**Fig. 1:**
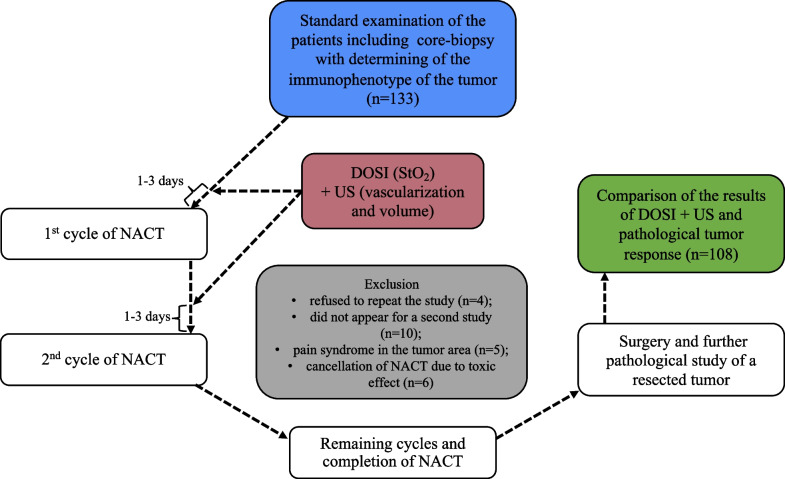
Study design

Before treatment, all patients were examined in accordance with the guidelines of the European Society for Medical Oncology (ESMO) [42] including mammography, ultrasound examination, and core biopsy of the tumor which provided the tumor receptor status. Tumor oxygenation by DOSI, tumor volume by US and tumor vascularization by Doppler US were determined twice, 1–3 days before the start of NACT and 1–3 days before the second cycle of NACT. The average interval between the first and second cycles was 21 days (± 2 days). After NACT completion, patients underwent surgery followed by pathological examination and assessment of the pathological tumor response to treatment (Fig. 1).

### Histopathology

After surgery, the tumor was assigned a response status according to Miller–Payne scale [43] which implies the five grades depending on the residual number of viable tumor cells assessed after chemotherapy. For binary classification of response, an accurate evaluation with a calculation of the number of viable malignant cells was performed. In order to build a regression logistic model, we introduced a criterion for a “high” response (reducing the number of viable tumor cells by more than 50% or “low” response (reducing the number of viable tumor cells by less than 50%).

### Imaging procedures

#### Doppler US

**Fig. 2:**
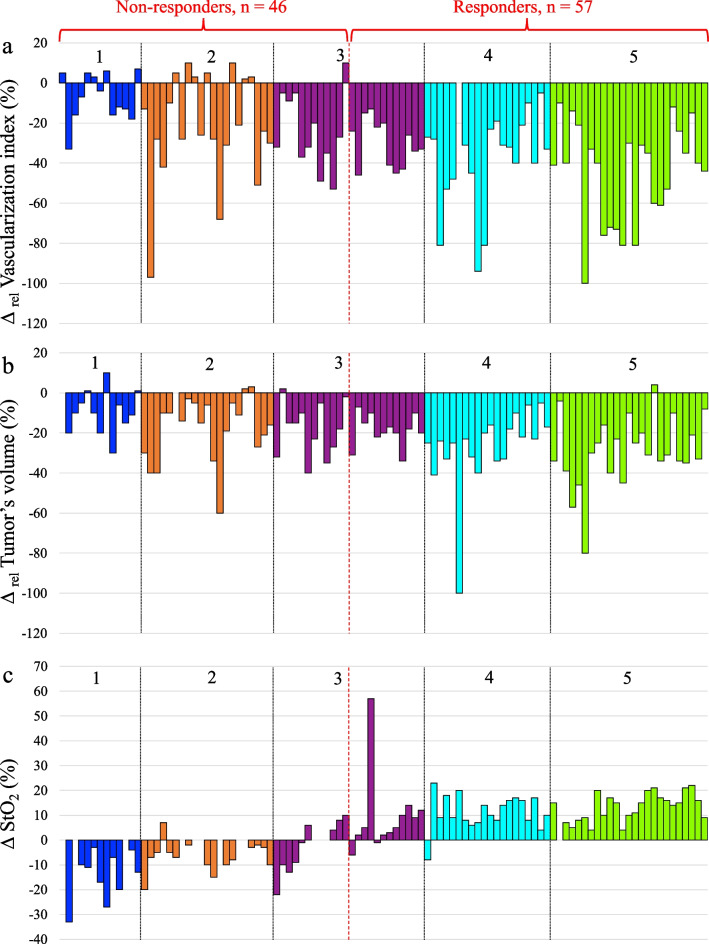
Changes in the tumor oxygen saturation, tumor vascularization, and tumor volume of patient B, aged 33. Diagnosis: multifocal (2 nodes) right BC, stage IIa (T2mN0M0), invasive breast carcinoma of no special type G3 (**d**, × 100) HER2neu-positive cancer (estrogen receptors = 0 points, progesterone receptors = 0 points, Ki67 = 60% (**e**, × 100)). After 1 cycle of NACT, DOSI showed an increase in the oxygenation level by 10% (**a**). US revealed a 10% decrease in tumor volume (**b**) as well as a 30% decrease in tumor vascularization (**c**). A pathologic investigation after six NACT cycles and surgery failed to find vital tumor cells, equivalent to the 5 grade of pathological tumor response (**f**, × 100)

Scanning of the area of interest which was chosen to fully cover the tumor with proper margins was carried out by a Medison Accuvix-V20 US scanner (Samsung, Korea) by a 5.0–13.0 MHz multifrequency linear sensor in power Doppler mode. An original ultrasound technique was developed to assess breast tumor vascularization [44]. We calculated the average number of colored pixels (corresponding to the vessels) relative to the total number of pixels in all the images—a vascularization index (Fig. 2c). Then the difference between the two values (ΔVascularization index) was calculated. To assess the tumor blood flow changes during the treatment, we entered the value of Δ_rel_(relative) of the vascularization index (Δ_rel_ Vascularization index = ΔVascularization index × 100%/Vascularization index before the NACT).

#### Tumor volume

Tumor volume was determined automatically using the calculation options of the Medison Accuvix-V20 by the following formula:

Tumor volume (cm^3^) = length × width × thickness × ellipsoid correction factor (0.479)

It was measured before the start of the treatment and before the second cycle of NACT. Then, the difference between the two values was calculated, i.e., ΔTumor volume was determined. To assess the tumor volume changes during the treatment, we entered the value of Δ_rel_(relative) of the tumor volume (Δ_rel_ Tumor volume = ΔTumor volume × 100%/Tumor volume before the NACT) (Fig. 2b).

#### Diffuse optical spectroscopy imaging

DOSI was performed on the setup (Institute of Applied Physics RAS, Nizhny Novgorod, Russia) [45] utilizing a frequency-domain approach and parallel plane trans-illumination scanning geometry. Radiation from three lasers is coupled in a single fiber bundle which illuminates the studied volume at three wavelengths: 684 nm corresponding to the maximum absorption of deoxygenated hemoglobin, 850 nm corresponding to the maximum absorption of oxyhemoglobin, and 794 nm at which absorption coefficients of oxygenated and deoxygenated hemoglobin coincide. The images were obtained by simultaneous scanning by moving the source and the detector located along the sagittal axis from the opposite sides of the studied subject with a step of 1–2 mm synchronously. In each position, data were detected from all three sources. High-frequency modulation of the laser radiation intensity 140 MHz was used to evaluate reduced scattering and absorption coefficient independently, thus improving the quantitative data on concentrations of oxy- and deoxyhemoglobin.

#### Image analysis

The numerical processing of DOSI images was described in our previous study [44]. The tumor region was selected manually by combining the tumor area on mammograms and DOSI images, as well as taking into account ultrasound data. Tumor chromophore concentrations were calculated by taking the mean over the tumor ROI. Blood oxygen saturation was calculated as StO_2_ = [HbO_2_]/[HbO_2_ + HHb] × 100% where HbO_2_ and HHb are the concentrations of oxy- and deoxyhemoglobin reconstructed from absorption coefficients at three wavelengths. StO_2_ was calculated for each spatial position, thus creating a DOSI image (optical mammogram) as a 2-D map of tissue oxygenation in the scanning area. The oxygen state of tumor tissue was calculated from the DOSI image by averaging StO_2_ in the tumor area using the ImageJ program (NIH, USA) (Fig. 2a). StO_2_ was calculated twice before the first and before the second cycle of NACT. The difference between the two values (ΔStO_2_) was determined and compared with tumor response to treatment.

### Statistical analysis

For statistical data processing, the SPSS Statistics (v. 27) software package was used. The distributions were checked for normality using the Shapiro–Wilk test. Since there was no normal distribution of data, the nonparametric Spearman correlation coefficient was used. The bond strength was assessed using the Chaddock scale. For statistical modeling of the probability of an event, the logit model (binary logistic model) was used.

Separate models were run on each of three indicators: ΔStO_2_, Δ_rel_ Vascularization index, and Δ_rel_ Tumor volume. The fact of tumor response (decrease in the number of tumor cells after NACT by more than 50%—a “high” response) was taken as a binary event.

Besides, two supplementary models for ΔStO_2_ were developed for the prediction of the tumor complete response (fifth grade of PTR as a binary event) [see Additional file 1]. Model 4 was created for predicting the pCR in patients with triple-negative and HER2-positive breast cancer and Model 5 for predicting the pCR in all 103 patients included in the study.

The probability of an event occurring for a particular case was calculated by the formula:

$$p = \frac{1}{{1 + e^{{ - z}} }}$$

where $$z=b\times X+a$$

*X*—independent variable value, *a*, *b*—regression coefficients.

With a *p* value of less than 0.5, it can be assumed that the event will not occur (Low response); otherwise, an event is expected (High response).

The critical significance level was taken as *p* ≤ 0.05.

To assess the predictive value of ΔStO_2_, Δ_rel_Vascularization index, Δ_rel_ Tumor volume to the early BC response to NACT, the sensitivity, specificity, and accuracy of the methods were calculated according to the logit model.

## Results

After the first cycle of NACT, five patients showed a significant (more than 100% compared to baseline) increase in the number of tumor vessels. We assumed this was associated with severe peritumoral inflammation around necrotic areas, and therefore these patients were not included in the final analysis. The characteristics of all analyzed subjects (103 patients included in further analysis) are given in Table 1. When studying the pathologic tumor response (PTR), the first grade was detected in 13 patients (13%), the second grade in 21 patients (20%), the third grade in 24 patients (23%), and the fourth grade in 20 patients (20%). The fifth grade (pCR) was detected in 25 patients (24%). In accordance with the proposed criterion for tumor response (> 50% or < 50% of cells loss), 57 (55%) patients demonstrated a “high” response to treatment and 46 (45%) patients demonstrated a “low” response.

**Table 1:** Patients’ characteristics

Description		Number of patients (*n*) (*P* ± *σp*%)
Age	< 40	29 (27 ± 4.3%)
	41–50	31 (30 ± 4.3%)
	51–60	24 (24 ± 4.1%)
	> 61	19 (19 ± 3.7%)
BC stage	IIa	26 (25 ± 4.1%)
	IIb	44 (42 ± 4.7%)
	IIIa	16 (16 ± 3.5%)
	IIIb	12 (12 ± 3.1%)
	IIIc	5 (5 ± 2.1%)
State of regional lymph nodes	No metastases	33 (32 ± 4.5%)
	With metastases	70 (68 ± 4.5%)
Immunophenotype	Luminal A	12 (12 ± 3.5%)
	Luminal B HER2neu-negative	26 (25 ± 4.1%)
	Luminal B HER2neu-positive	12 (12 ± 9.5%)
	HER2neu-positive	18 (18 ± 3.6%)
	Triple negative	35 (33 ± 4.5%)
Scheme of NACT	Anthracyclines	39 (41 ± 4.7%)
	Anthracyclines and taxane	38 (35 ± 4.5%)
	Anthracyclines and trastuzumab	25 (23 ± 4.0%)
	Trastuzumab/pertuzumab	1 (1 ± 0.94%)
Grade of pathological tumor response (Miller and Payne scale)	1	13 (12 ± 3.1%)
	2	21 (21 ± 3.9%)
	3	24 (24 ± 4.1%)
	4	20 (19 ± 3.7%)
	5	25 (24 ± 4.1%)
Pathological tumor response (actual morphometric scale in our study)	High response	57 (55 ± 4.5%)
	Low response	46 (45 ± 4.5%)
Total		103 (100%)

### Doppler US

**Fig. 3:**
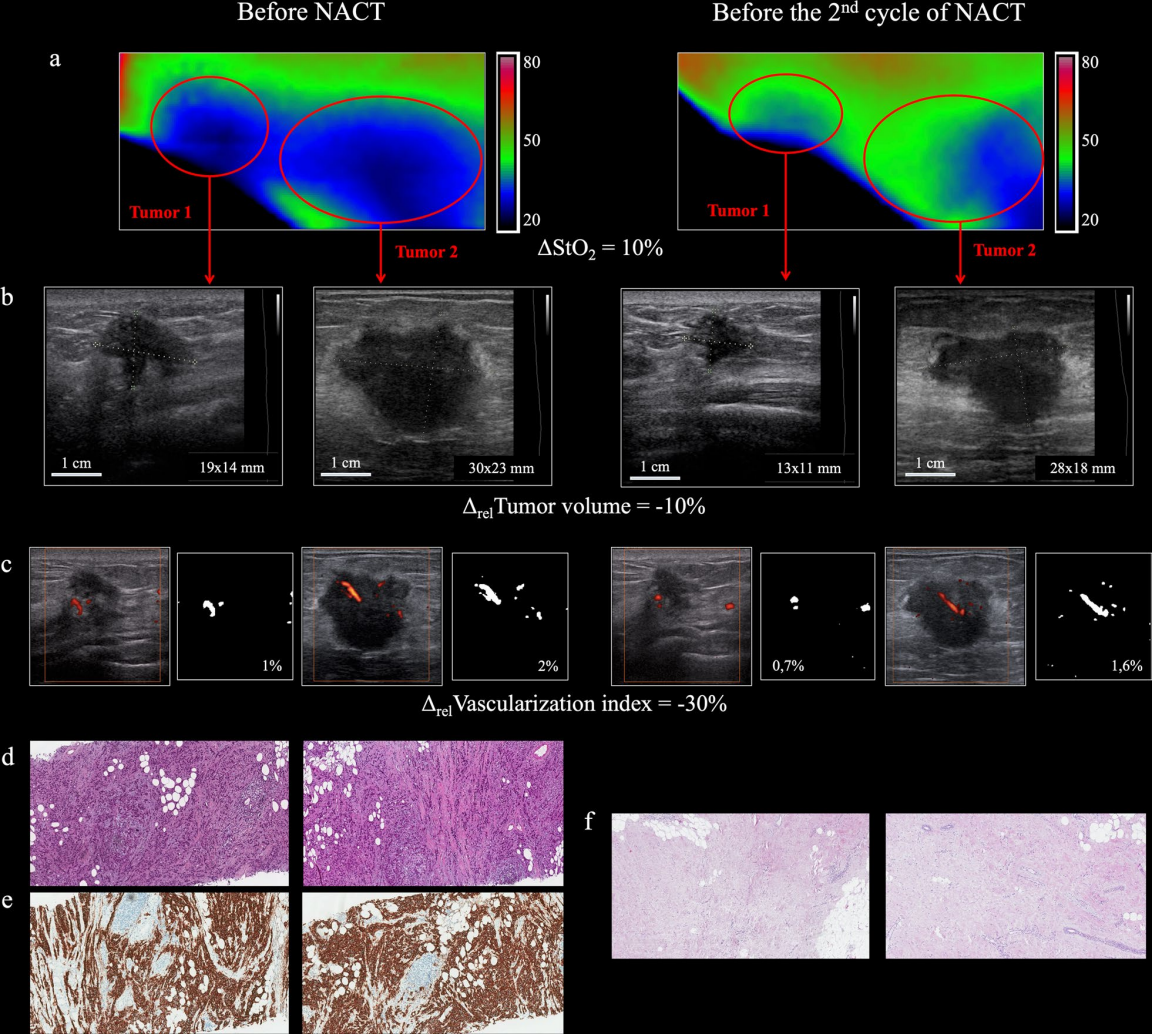
Changes in tumor vascularization (**a**), tumor volume (**b**), and tumor oxygenation level (**c**) before NACT and before the 2nd cycle of NACT, depending on the PTR (*n* = 103)

In 46 patients who demonstrated a “low” response, a decrease in the number of tumor vessels was observed in 33 (72%), and an increase in 13 (28%) (Fig. 3a). Among 57 “high” responders, 56 showed a decrease in tumor vascularization. Statistical analysis showed a moderate negative correlation between Δ_rel_Vascularization index and PTR (*ρ* = − 0.502, *p* ≤ 0.01).

### Tumor volume

In the vast majority (94 patients, 91%), a decrease in tumor volume has been observed after the first cycle of NACT (Fig. 3b). Among 46 patients with a “low” response, a tumor volume increased in 6 cases and has not changed in 2. Among 57 patients with a “high” response, 56 patients showed a decrease in the tumor volume. A moderate negative correlation was revealed between Δ_rel_ Tumor volume and PTR (*ρ* = − 0.416, *p* ≤ 0.01).

### Diffuse optical spectroscopy imaging

Breast tumors showed multidirectional dynamics of oxygenation depending on the PTR. Among 57 patients with a “high” response, 53 (93%) showed an increase in StO_2_ after the 1st cycle of NACT. The oxygenation decreased in three patients, but in one it did not change (Fig. 3c). Twenty-nine of the 46 patients with a “low” response experienced a decrease in tumor oxygenation after the 1st cycle of NACT. In 12 cases, the level of oxygenation did not change, and in 5 cases it increased. A positive strong correlation was found between the change in the oxygenation (ΔStO_2_) and the PTR (*ρ* = 0.802, *p* ≤ 0.01).

### Binary logistic model

#### Model 1 "Δ_rel_ Tumor volume"

The overall number of patients correctly assigned to the groups of "high” response and "low” response using the variable "Δ_rel_ Tumor volume" was 70.9% (Table 2).

**Table 2:** Classification for Model 1 (“Δ_rel_Tumor volume”)

Observed	Predicted		Percentage of correct
	Low response	High response	
Low response (*n* = 46)	28	18	60.9
High response (*n* = 57)	12	45	78.9
Total	70.9		

The sensitivity, specificity, and accuracy of the index for predicting PTR to NACT were 78.9%, 60.9%, and 70.9%, respectively.

Despite the presence of a fairly large number of false positive cases, all variables in the regression equation appeared to be statistically significant (Table 3): *a* = − 0.831 (*p* = 0.027) и *b* = − 0.052 (*p* = 0.001).

$$z = ~ - 0.0{\text{52}} \times \left( {\Delta _{{{\text{rel}}}} {\text{Tumor}}\;{\text{volume}}} \right)\;{-}\;0.{\text{831}}\;\left( {{\text{Model}}\;{\text{1}}\;"\Delta _{{{\text{rel}}}} {\text{Tumor volume"}}} \right)$$

**Table 3:** Variables in Model 1 equation (“Δ_rel_ Tumor volume”)

Variable	Value	Standard deviation	Significance level *p*
*b*	− 0.052	0.016	0.001
*a*	− 0.831	0.376	0.027

To test Model 1, a patient was randomly selected with “Δ_rel_Tumor volume” = -3.

$$z = - 0.052 \times \left( { - 3} \right){-} \, 0.831 = - 0.675$$

$$p = \frac{1}{{1 + e^{{ - \left( { - 0.675} \right)}} }} = 0.337$$

According to the model, the patient will respond to treatment with a probability of 33.7%. This was confirmed by a pathomorphological tumor study of the residual tumor (the number of viable tumor cells was 90% which corresponds to the second grade of PTR).

#### Model 2 "Δ_rel_Vascularization index"

When using the "Δ_rel_ Vascularization index" model, the total percentage of correct predictions decreases up to 68% (Table 4).

**Table 4:** Model 2 classification (“Δ_rel_Vascularization index”)

Observed	Predicted		Percentage of correct
	Low response	High response	
Low response (*n* = 46)	27	19	58.7
High response (*n* = 57)	14	43	75.4
Total	68.0		

The sensitivity, specificity, and accuracy of the indicator Δ_rel_Vascularization index for predicting PTR were 75.4%, 58.7%, and 68.0%, respectively.

In Model 2 (Table 5), only the variable *b* = − 0.043 (*p* = 0.001) turned out to be statistically significant. For the constant *a* = − 0.989, the critical level of significance was not overcome (*p* = 0.07), so it is not advisable to test Model 2.

**Table 5:** Variables in Model 2 equation (“Δ_rel_Vascularization index”)

Variable	Value	Standard deviation	Significance level *p*
b	− 0.043	0.011	0.001
a	− 0.989	0.366	0.070

*z* = − 0.043 × X(Δ_rel_ Vascularization index)—0.989 (Model 2 (“Δ_rel_ Vascularization index”)).

#### Model 3 "ΔStO_2_"

The last step of the regression analysis was the introduction of the variable “the oxygenation level” (ΔStO_2_) into the model.

The “ΔStO_2_” variable significantly increases the number of correct predictions of low response to treatment. The overall percentage of correct predictions was 89.3% (Table 6). Early changes in the tumor oxygenation for prediction of the PTR demonstrated high sensitivity, specificity, and accuracy—89.5%, 89.1%, and 89.3%, respectively.

**Table 6:** Model 2 classification (“ΔStO_2_”)

Observed	Predicted		Percentage of correct
	Low response	High response	
Low response (*n* = 46)	41	5	89.1
High response (*n* = 57)	6	51	89.5
Total	89.3		

Both variables of the regression equation turned out to be statistically significant: *a* = − 0.773 (*p* = 0.035) and *b* = 0.318 (*p* = 0.001) (Table 7).

**Table 7:** Variables in Model 3 equation (“ΔStO_2_”)

Variable	Value	Standard deviation	Significance level *p*
*b*	0.318	0.064	0.001
*a*	− 0.773	0.367	0.035

*z* = 0.318 × X(ΔStO_2_) − 0.773 (Model 3 “ΔStO_2_”)

To test the oxygenation level model, we have randomly used data from patients from the two groups.

Patient 1 (ΔStO_2_ = − 15)

*z* = 0.318 × ( − 15) − 0.773 = − 4.91

$$p = \frac{1}{{1 + e^{{ - \left( { - 4.91} \right)}} }} = 0.0073$$

This patient will have a low response to treatment with a probability of 99.27%, which was confirmed by a pathological tumor response (the number of viable malignant cells was 94% which corresponded to the second grade of PTR).

Patient 2 (ΔStO_2_ = 18)

*z* = 0.318 × (18) − 0.773 = 4.95

$$p = \frac{1}{{1 + e^{{ - 4.95}} }} = 0.993$$

The second patient with a probability of 99.3% could be attributed to the high response group, which was confirmed by the fifth grade of PTR.

Thus, Model 1 "Δ_rel_ Tumor volume" and Model 2 "Δ_rel_ Vascularization index" have the same limitation: they do not accurately predict a lack of response to treatment, while predicting a positive response is relatively good. The combined inclusion of all of the above variables (ΔStO_2_, Δ_rel_ Vascularization index, and Δ_rel_ Tumor volume) into the model did not show an improvement in the predictive values.

According to Model 4, the sensitivity, specificity, and accuracy of the «ΔStO2» indicator for predicting pCR in patients with triple-negative and HER2-positive breast cancer were 60.9%, 80.9%, and 74.3%, respectively (65 patients). According to Model 5, the sensitivity, specificity, and accuracy of the ΔStO2 indicator for predicting pCR were 20%, 95.2%, and 77.8%, respectively (103 patients). A detailed description of Model 4 and Model 5 is presented in Additional file 1.

### Discussion

Starting the study of tumor oxygen state in the course of chemotherapy, we proceeded to that the effects of chemotherapy on tumors are due to both direct death of tumor cells and the delicate interplay between tumor vasculature responses and the inextricably linked changes in oxygen state [see Additional file 2] as well as its contribution to the processes driving the tumor cells death [46, 47]*.* Accordingly, for the development of criteria predicting the sensitivity of breast tumors to chemotherapy, we used complementary methods: assessment of the dynamics of tumor volume and blood flow using US and the dynamics of tumor oxygenation by DOSI.

Our study confirmed that ΔStO_2_ correctly reflects tumor changes under the “pressure” of cytostatic therapy 3 weeks after treatment starts. The sensitivity, specificity, and accuracy of this indicator in relation to predicting tumor response appeared to be 89.5%, 89.1%, and 89.3%, respectively. Changes in oxygenation when constructing a logistic regression (Model 3) made it possible to attribute patients to the group of both “high response” and “low response” (89.3%) with a high probability and predicting the response with a high accuracy when validating the model. Similar results were obtained in [48]*,* where the use of logistic regression based on the ratio of saturation of normal and tumor tissues (z-score) within 10 days after the start of treatment turned out to be a significant predictor of the complete response. The main parameter reflecting the sensitivity of the tumor to chemotherapy was the improvement in the oxygenation of the neoplasm after the chemotherapy administration. Apparently, this appears to be due to a well-known radiation biology phenomenon of reoxygenation as a result of a decrease in oxygen consumption by damaged tumor cells [49, 50].

The study of the vascular bed of the tumor using the power Doppler method revealed a decrease in the number of tumor vessels after the first cycle of chemotherapy in the vast majority of cases. All the tumors with a “high” response to treatment demonstrated a decrease in the vascularization of the neoplasm. In general, this indicator demonstrated relatively high sensitivity (75.4%), but low specificity (58.7%) and low accuracy (68.0%). This may be explained by the fact that tumors contain predominantly small (less than 100 µm) and immature blood vessels with low blood flow velocity [35]. Apparently, the resolution of power Doppler ultrasound is not sufficient to identify such vessels and correctly assess the blood filling of the tumor tissue and its changes in the course of chemotherapy. The study demonstrated the limitations of noninvasive imaging biomarkers of breast cancer response, when tumors with extended necrotic areas cannot be evaluated correctly because of unpredictable vessel’s reaction.

The change in the volume of tumor tissue also did not demonstrate a high predictive ability in relation to the pathological tumor response (70.9% of correct predictions). However, in the case of “low” response, the vast majority of tumors showed a decrease in tumor volume after the first cycle of NACT. It can be assumed that in this case, chemotherapy reduces the inflammatory component of the tumor or death of the drug-sensitive fraction of tumor cells occurs.

In an earlier work [44], we concluded that it is necessary to calculate an accurate number of the residual viable tumor cells after NACT in order to study in more detail the changes in grade 3 of PTR tumors. We used a loss of 50% cellularity as a threshold, and not a standard histopathological score, since a pronounced heterogeneity of changes, which are attributed to the third grade of tumor response. It includes tumors with a loss of cellularity in the range from 30 to 90%, in which multidirectional changes in oxygenation were detected (Fig. 3). From this point of view, our approach (an accurate calculation of cell loss) seems to be more reasonable than a formalized assignment a tumor to any grade of pathological response. A similar approach was used by [30]*,* where the 50% change in the volume of the tumor node was chosen as a criterion of response.

Our study did not allow for identifying tumors that completely responded to treatment with sufficient accuracy. Model 5, where a pCR was chosen as a binary event, showed only 20% correct predictions of complete response. A low ability of the method to predict a complete response is its characteristic feature, since it is very difficult to separate grade 4 (10% of viable tumor cells) and grade 5 (0% of viable tumor cells) in the early time after the start of a treatment. Our approach does not allow capturing such minor differences; however, it makes it possible to predict the trend of tumor resistance to treatment very well, which is also very important information regarding escalation or de-escalation of therapy, also a need of additional genetic study of the tumor.

## Conclusion

We observed changes in all three parameters (oxygenation, tumor volume, and blood flow) at the early time after the start of the treatment. However, a high predictive potential for early assessment of tumor sensitivity to NACT was found only in changes in the level of oxygenation, ΔStO_2_, (*ρ* = 0.802, *p* ≤ 0.01). The regression model can be used for the prediction of the response to treatment with a high probability of a correct conclusion. The analysis showed that the “Tumor volume” model and the “Vascularization index” model did not accurately predict the absence of a pathological tumor response to treatment (60.9 and 58.7%, respectively), while predicting a positive response to treatment was relatively better (78.9 and 75.4%, respectively).

## Supplementary Information

**Additional file 1.** Binary logistic models  for prediction the tumor complete response (the 5th grade of PTR as a binary event) using ΔStO2 indicator.

**Additional file 2.** Oxyhemoglobin and deoxyhemoglobin tumor concentration before NACT and before the 2nd cycle of NACT (103 patients).

## Data Availability

These data that support the findings of this manuscript can be made available from the corresponding author upon reasonable request.

## Abbreviations

BC: Breast cancer
DOSI: Diffuse optical spectroscopy imaging
ECOG: Eastern cooperative oncology group
HER2neu: Human epidermal growth factor receptor-2
HbO_2_: Oxyhemoglobin
HHb: Deoxyhemoglobin
NACT: Neoadjuvant chemotherapy
pCR: Pathologic complete response
PTR: Pathological tumor response
Rel: Relative
StO_2_: Oxygen saturation
US: Ultrasound
ROI: Region of interest
