# Correction: Changes in the tumor oxygenation but not in the tumor volume and tumor vascularization reflect early response of breast cancer to neoadjuvant chemotherapy

**DOI:** 10.1186/s13058-023-01614-7

**Published:** 2023-02-07

**Authors:** Mikhail V. Pavlov, Anna P. Bavrina, Vladimir I. Plekhanov, German Yu. Golubyatnikov, Anna G. Orlova, Pavel V. Subochev, Diana A. Davydova, Ilya V. Turchin, Anna V. Maslennikova

**Affiliations:** 1 Nizhny Novgorod Regional Clinical Oncology Dispensary, Delovaya St., 11/1, Nizhny Novgorod, Russia 603126; 2 Privolzhsky Research Medical University, Minina Square, 10/1, Nizhny Novgorod, Russia 603950; 3 Institute of Applied Physics RAS, Ul’yanov Street, 46, Nizhny Novgorod, Russia 603950; 4 National Research Lobachevsky State University of Nizhny Novgorod, Gagarin Ave., 23, Nizhny Novgorod, Russia 603022

**Correction to: Breast Cancer Research (2023) 25:12**

**https://doi.org/10.1186/s13058-023-01607-6**

Following publication of the original article [1], it was noted that due to a typesetting error Figs. 2 and 3 were reversed. The figure legends are both correct and in the right place.

Figure 2 should be Fig. 3.

Figure 3 should be Fig. 2.

The correct figure placements have been included in this correction, and the original article has been corrected.

The original article [1] has been updated.
