# Supplementary material for: Changes in the tumor oxygenation but not in the tumor volume and tumor vascularization reflect early response of breast cancer to neoadjuvant chemotherapy
Source: Breast Cancer Res. 2023 Jan 30;25:12. doi: 10.1186/s13058-023-01607-6 (PMC9887770; doi:10.1186/s13058-023-01607-6)
Supplement: Supplementary file 1 — Additional file 1. Binary logistic models for prediction the tumor complete response (the 5th grade of PTR as a binary event) using ΔStO2 indicator. [file 13058_2023_1607_MOESM1_ESM.docx]

**Model 4.** "ΔStO_2_" for prediction pCR (5^th^ grade of PTR) in patients with triple-negative and HER2-positive breast cancer (65 patients).

Table S1. Model 4 Classification Table (“ΔStO_2_”).

| Observed | Predicted | | Percentage of correct |
| --- | --- | --- | --- |
|  | Non-responders | Responders |  |
| Non-responders (n=42) | 35 | 7 | 80.9 |
| Responders (n=23) | 9 | 14 | 60.9 |
| Total | 74.3 | | |

Table S2. Variables in Model 4 Equation (“ΔStO2”).

| Variable | Value | Standard deviation | Significance level p |
| --- | --- | --- | --- |
| b | 0.157 | 0.042 | <0.001 |
| a | -2.071 | 0.532 | <0.001 |

Both regression coefficients were statistically significant: (Table S2):  а = -2.071 (р<0.001) and b = 0.157 (р<0.001).

z = 0.157×Х(ΔStO_2_) - 2.071 (Model 4 (“ΔStO_2_”)).

Patient 1 (ΔStO_2_ = -13).

z = 0.157×(-13) – 2.071 = -4.112

$$р=\frac{1}{1+e^{-(-4.112)}}=0.016$$

According to the model the patient will respond to treatment with a probability of 1.6%. This was confirmed by a pathomorphological study of the residual tumor (3rd grade of PTR).

Patient 2 (ΔStO_2_ = 17).

z = 0.157×(17) – 2.071 = 0.598

$$р=\frac{1}{1+e^{-0.598}}=0.645$$

The second patient with a probability of 64.5% could be attributed to the responders group, which was confirmed by a 5^th^ grade of PTR.

**Model 5.** "ΔStO_2_" for prediction pCR (5^th^ grade of PTR) in all 103 patient including in the study.

Table S3. Model 5 Classification Table (“ΔStO_2_”).

| Observed | Predicted | | Percentage of correct |
| --- | --- | --- | --- |
|  | Non-responders | Responders |  |
| Non-responders (n=78) | 75 | 3 | 95.2 |
| Responders (n=25) | 20 | 5 | 20.0 |
| Total | 77.8 | | |

Although both regression coefficients again turned out to be statistically significant (Table S4), a large number of false negative results were noteworthy (Table S3). The percentage of correctly identified patients in the responders group was only 20%. Together, Model 5 showed a high percentage of correct assignment to the group, non-responders – 95.2%. It can be assumed that Model 5 will have a drawback in identifying patients in the responders group.

Table S4. Variables in Model 5 Equation (“ΔStO2”).

| Variable | Value | Standard deviation | Significance level p |
| --- | --- | --- | --- |
| b | 0.111 | 0.030 | 0.001 |
| a | -1.963 | 0.374 | 0.001 |

z = 0.111×Х(ΔStO_2_) - 1.963(Model 5 (“ΔStO_2_”)).

Patient 1 (ΔStO_2_ = -13).

z = 0.111×(-13) – 1.963 = -3.41

$$р=\frac{1}{1+e^{-(-3.41)}}=0.032$$

This patient will be in responders group to treatment with a probability of 3.2%, which was confirmed by a pathological tumor response (number of viable malignant cells was 100% which corresponded to the 1st grade of PTR).

Patient 2 (ΔStO_2_ = 17).

z = 0.111×(17) – 1.963= -0.076

$$р=\frac{1}{1+e^{-0.076}}=0.481$$

For the second patient with pCR (5th grade of PTR), the probability of being assigned to the responders group was 48.1 %. Therefore, it can be concluded that Model 5 had insufficient power to identify patients from the responders group. At the same time, Model 5 clearly identified patients in the non-responders group. These calculations confirmed the conclusions presented for Table S3.
