# Supplementary material for: Changes in the tumor oxygenation but not in the tumor volume and tumor vascularization reflect early response of breast cancer to neoadjuvant chemotherapy
Source: Breast Cancer Res. 2023 Jan 30;25:12. doi: 10.1186/s13058-023-01607-6 (PMC9887770; doi:10.1186/s13058-023-01607-6)
Supplement: Supplementary file 2 — Additional file 2. Oxyhemoglobin and deoxyhemoglobin tumor concentration before NACT and before the 2nd cycle of NACT (103 patients). [file 13058_2023_1607_MOESM2_ESM.docx]

Table S5. Oxyhemoglobin and deoxyhemoglobin concentrations before NACT and before the 2nd cycle of NACT (103 patients).

|  | Oxyhemoglobin (HbO_2_) | | Deoxyhemoglobin (HHb) | |
| --- | --- | --- | --- | --- |
|  | Before NACT | After first cycle NACT | Before NACT | After first cycle NACT |
| 1 grade of pathological tumor response (Miller-Payne) | | | | |
| #1 | 9.80 | 9.50 | 29.24 | 27.90 |
| #2 | 7.39 | 3.98 | 10.72 | 49.73 |
| #3 | 5.50 | 5.30 | 9.90 | 9.80 |
| #4 | 6.20 | 4.54 | 11.20 | 12.65 |
| #5 | 7.40 | 5.13 | 12.80 | 14.69 |
| #6 | 6.84 | 6.12 | 13.80 | 14.46 |
| #7 | 7.45 | 7.31 | 8.48 | 17.32 |
| #8 | 9.99 | 6.12 | 8.27 | 15.74 |
| #9 | 9.14 | 7.45 | 17.07 | 19.81 |
| #10 | 8.34 | 4.36 | 12.51 | 17.92 |
| #11 | 9.01 | 8.89 | 23.95 | 23.38 |
| #12 | 4.69 | 4.19 | 8.17 | 8.55 |
| #13 | 6.79 | 4.58 | 10.24 | 12.35 |
| 2 grade of pathological tumor response (Miller-Payne) | | | | |
| #14 | 15.60 | 22.56 | 23.25 | 87.34 |
| #15 | 28.30 | 13.50 | 54.33 | 36.31 |
| #16 | 10.10 | 15.14 | 18.46 | 35.21 |
| #17 | 19.30 | 21.80 | 50.50 | 40.65 |
| #18 | 33.30 | 32.80 | 59.08 | 71.81 |
| #19 | 10.90 | 15.80 | 18.05 | 36.17 |
| #20 | 7.80 | 14.80 | 16.84 | 31.43 |
| #21 | 15.60 | 14.20 | 38.77 | 39.87 |
| #22 | 13.40 | 13.00 | 31.79 | 30.67 |
| #23 | 27.98 | 25.43 | 82.35 | 73.52 |
| #24 | 23.45 | 26.65 | 35.20 | 63.57 |
| #25 | 15.60 | 19.65 | 26.43 | 69.10 |
| #26 | 38.50 | 34.70 | 56.92 | 50.56 |
| #27 | 26.45 | 25.84 | 40.58 | 63.11 |
| #28 | 14.69 | 28.34 | 29.60 | 84.16 |
| #29 | 25.45 | 24.69 | 59.90 | 57.12 |
| #30 | 36.41 | 37.48 | 60.53 | 62.28 |
| #31 | 12.65 | 10.62 | 31.47 | 31.46 |
| #32 | 28.45 | 26.54 | 44.89 | 44.98 |
| #33 | 5.34 | 11.36 | 15.72 | 40.39 |
| #34 | 9.99 | 8.43 | 15.92 | 21.07 |
| 3 grade of pathological tumor response (Miller-Payne) | | | | |
| #35 | 16.48 | 18.32 | 25.61 | 89.25 |
| #36 | 24.90 | 0.00 | 43.40 | 0.00 |
| #37 | 8.64 | 8.43 | 13.77 | 24.13 |
| #38 | 50.14 | 41.69 | 76.60 | 96.68 |
| #39 | 34.85 | 30.14 | 51.07 | 45.59 |
| #40 | 14.69 | 16.58 | 31.86 | 27.17 |
| #41 | 25.36 | 28.93 | 66.66 | 75.51 |
| #42 | 21.45 | 34.16 | 37.22 | 58.39 |
| #43 | 34.10 | 45.35 | 68.12 | 88.66 |
| #44 | 25.30 | 18.50 | 69.85 | 42.68 |
| #45 | 11.10 | 16.30 | 32.12 | 31.86 |
| #46 | 31.33 | 45.35 | 62.33 | 59.34 |
| #47 | 17.60 | 15.30 | 33.49 | 38.48 |
| #48 | 12.54 | 21.65 | 45.73 | 68.75 |
| #49 | 32.65 | 45.65 | 65.21 | 74.70 |
| #50 | 0.20 | 4.00 | 55.36 | 2.95 |
| #51 | 28.80 | 22.10 | 79.28 | 64.07 |
| #52 | 41.64 | 45.35 | 66.91 | 65.88 |
| #53 | 16.47 | 11.84 | 36.07 | 22.24 |
| #54 | 34.17 | 22.47 | 62.46 | 33.63 |
| #55 | 21.65 | 25.31 | 60.16 | 43.32 |
| #56 | 21.65 | 12.47 | 58.18 | 18.09 |
| #57 | 26.65 | 32.47 | 74.07 | 59.71 |
| #58 | 14.65 | 6.65 | 42.05 | 10.74 |
| 4 grade of pathological tumor response (Miller-Payne) | | | | |
| #59 | 46.64 | 35.62 | 75.61 | 82.52 |
| #60 | 4.00 | 9.30 | 12.20 | 10.20 |
| #61 | 31.65 | 32.47 | 78.86 | 53.79 |
| #62 | 21.90 | 15.87 | 64.45 | 20.44 |
| #63 | 14.00 | 20.30 | 33.36 | 32.79 |
| #64 | 15.50 | 17.70 | 50.29 | 22.55 |
| #65 | 12.50 | 16.60 | 26.39 | 24.39 |
| #66 | 24.60 | 22.40 | 58.36 | 40.96 |
| #67 | 12.60 | 16.00 | 37.86 | 33.82 |
| #68 | 6.46 | 6.31 | 37.61 | 15.71 |
| #69 | 33.31 | 32.47 | 85.11 | 52.68 |
| #70 | 35.51 | 32.67 | 77.05 | 49.94 |
| #71 | 28.21 | 30.15 | 77.64 | 44.02 |
| #72 | 15.47 | 5.47 | 50.23 | 8.36 |
| #73 | 23.68 | 24.56 | 68.69 | 33.04 |
| #74 | 19.54 | 16.46 | 60.35 | 24.22 |
| #75 | 23.47 | 21.40 | 50.67 | 32.57 |
| #76 | 31.25 | 32.47 | 95.50 | 45.48 |
| #77 | 41.23 | 43.31 | 71.27 | 63.24 |
| #78 | 24.30 | 23.47 | 79.41 | 46.73 |
| 5 grade of pathological tumor response (Miller-Payne) | | | | |
| #79 | 22.17 | 33.31 | 64.28 | 48.41 |
| #80 | 15.41 | 13.51 | 47.11 | 40.62 |
| #81 | 25.51 | 28.51 | 56.23 | 45.78 |
| #82 | 10.45 | 9.65 | 20.29 | 15.18 |
| #83 | 28.65 | 34.65 | 84.37 | 68.87 |
| #84 | 16.30 | 15.50 | 38.69 | 24.61 |
| #85 | 41.40 | 21.80 | 77.92 | 34.53 |
| #86 | 13.68 | 15.47 | 40.30 | 18.71 |
| #87 | 13.90 | 15.50 | 23.03 | 16.80 |
| #88 | 15.30 | 25.80 | 41.41 | 33.34 |
| #89 | 25.55 | 26.65 | 75.23 | 40.00 |
| #90 | 13.80 | 6.30 | 28.47 | 10.72 |
| #91 | 21.30 | 4.90 | 55.62 | 8.25 |
| #92 | 11.50 | 20.40 | 34.56 | 35.93 |
| #93 | 28.46 | 34.65 | 83.99 | 51.05 |
| #94 | 19.53 | 24.31 | 72.98 | 34.16 |
| #95 | 14.00 | 22.65 | 55.20 | 31.86 |
| #96 | 34.37 | 52.47 | 86.87 | 63.58 |
| #97 | 32.68 | 34.10 | 99.15 | 50.62 |
| #98 | 23.35 | 19.65 | 64.90 | 28.78 |
| #99 | 23.00 | 32.61 | 53.35 | 40.53 |
| #100 | 17.53 | 51.31 | 72.53 | 74.16 |
| #101 | 24.56 | 16.51 | 72.32 | 18.55 |
| #102 | 5.13 | 2.55 | 15.77 | 3.75 |
| #103 | 36.68 | 36.02 | 80.02 | 52.31 |
|  |  |  |  |  |
|  |  |  |  |  |
|  |  |  |  |  |
|  |  |  |  |  |
|  |  |  |  |  |
|  |  |  |  |  |
|  |  |  |  |  |
|  |  |  |  |  |
|  |  |  |  |  |
|  |  |  |  |  |
